# Left bundle, right diagnosis

**DOI:** 10.1007/s12471-019-1255-0

**Published:** 2019-03-01

**Authors:** A. E. Schaafsma, C. Coolsma, H. Lameijer

**Affiliations:** Department of Emergency Medicine, Medical Centre Leeuwarden, Leeuwarden, The Netherlands

A 79-year-old male was brought to the emergency department by emergency medical services (EMS) with retrosternal chest pain radiating to both shoulders. The pain started 2 hours ago while he was working in his garden. His medical history included hypertension, hypercholesterolaemia and a transient ischaemic attack. Physical examination showed tachypnoea, 37 breaths per minute, without abnormalities at pulmonary or cardiac auscultation, a heart rate of 88 beats per minute, a blood pressure of 111/73 mm Hg and unremarkable findings on abdominal examination. However, the patient looked clammy and grey. Nitroglycerine (GNT) administered sublingually relieved the pain. A partial electrocardiogram was performed by an EMS nurse (Fig. 1). Which abnormalities raise your concern?

**Fig. 1:**
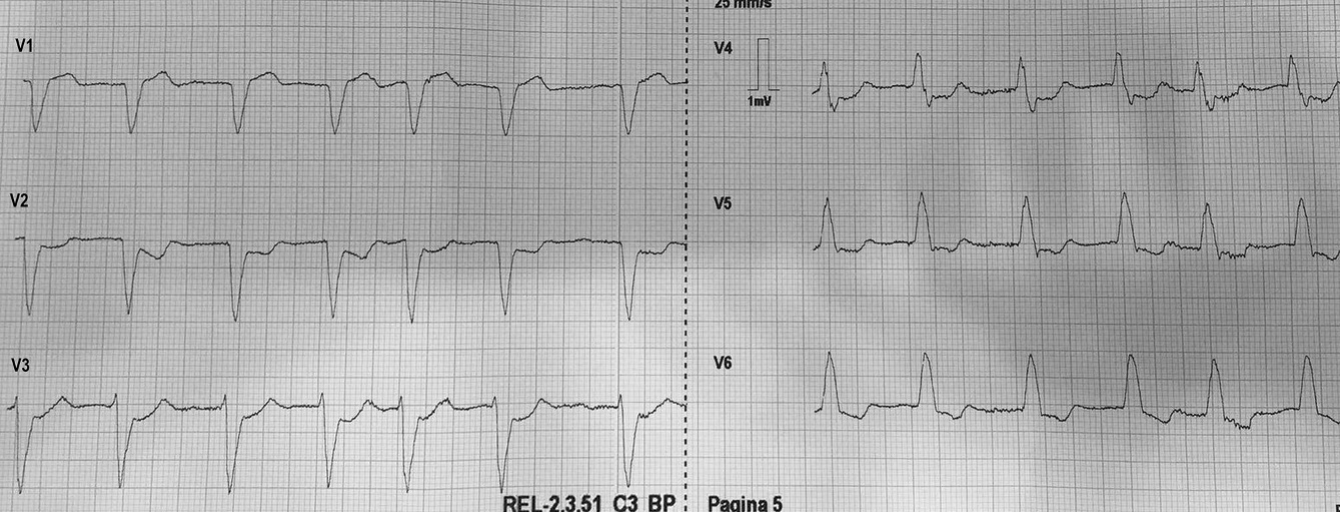
The anterior electrocardiogram leads at presentation performed by emergency medical services

A. A new left bundle branch block
B. The negative T wave in leads V5 and V6
C. The ST depression in leads V2–V4
D. The irregularity of the cardiac rhythm

## Answer

You will find the answer elsewhere in this issue.

